# Persistent Alopecia in a Breast Cancer Patient Following Taxane Chemotherapy and Adjuvant Endocrine Therapy: Case Report and Review of Post-treatment Hair Loss in Oncology Patients with Breast Cancer

**DOI:** 10.7759/cureus.3056

**Published:** 2018-07-27

**Authors:** Tyler Werbel, Philip R Cohen

**Affiliations:** 1 School of Medicine, University of California San Diego, San Diego, USA; 2 Dermatologist, San Diego Family Dermatology, San Diego, USA

**Keywords:** alopecia, breast, cancer, chemotherapy, endocrine, hair, loss, minoxidil, permanent, taxane

## Abstract

Taxane chemotherapy and adjuvant endocrine therapy are commonly used in breast cancer patients following surgery. We describe a 59-year-old woman with a triple-positive invasive right breast cancer that was treated with surgery, radiation, chemotherapy, and adjuvant hormonal therapy. She subsequently developed scalp alopecia, with histopathological features of both androgenetic alopecia and alopecia areata; the hair loss did not resolve after completion of her chemotherapy. Significant clinical improvement was observed with topical minoxidil therapy. PubMed was searched for the following terms: alopecia, breast, cancer, chemotherapy, endocrine, hair, loss, minoxidil, permanent, and taxane. The papers containing these terms and their references were reviewed. Temporary hair loss is frequently observed following taxane chemotherapy; however, albeit uncommon, persistent or permanent alopecia may occur in women with breast cancer who have been treated with taxane chemotherapy and endocrine therapy. It may be reasonable to initiate therapy with topical minoxidil in breast cancer patients who develop alopecia after treatment with either taxane chemotherapy or endocrine therapy alone or both.

## Introduction

Alopecia is a common adverse cutaneous event in oncology patients receiving antineoplastic therapy. Treatment-associated alopecia has been observed in patients receiving taxane chemotherapy, hormonal therapy, or both [1]. We describe a woman who developed prolonged alopecia following treatment which included docetaxel and subsequent hormonal therapy who subsequently experienced hair regrowth after treatment with topical minoxidil.

## Case presentation

A 59-year-old woman presented for evaluation of scalp alopecia. Her past medical history was significant for PT1cN1mi estrogen receptor (ER)+, progesterone receptor (PR)+, human epidermal growth factor receptor (HER)2+ g3 invasive ductal carcinoma of the right breast diagnosed 15 months earlier. She had been treated with bilateral lumpectomy with right-sided sentinel lymph node biopsy and started chemotherapy nine months earlier; she received pertuzumab, docetaxel, carboplatin, and trastuzumab every three weeks for six cycles and was maintained on trastuzumab 6 mg/kg every three weeks for one year. Three weeks after completing taxane chemotherapy, she began treatment with anastrozole 1 mg daily (which was switched to tamoxifen 20 mg daily due to joint pain). She was also treated with radiation therapy and is currently on neratinib 240 mg daily; neratinib is a tyrosine kinase inhibitor anticancer drug used to prevent recurrence in patients with early-stage HER2+ breast cancer who have finished at least one year of post-surgery trastuzumab therapy.

She noted hair loss beginning after her first course of systemic chemotherapy. It became more extensive throughout the remainder of her treatment. She had not experienced any regrowth of scalp hair since the completion of chemotherapy nor during her current hormonal therapy.

**Figure 1:**
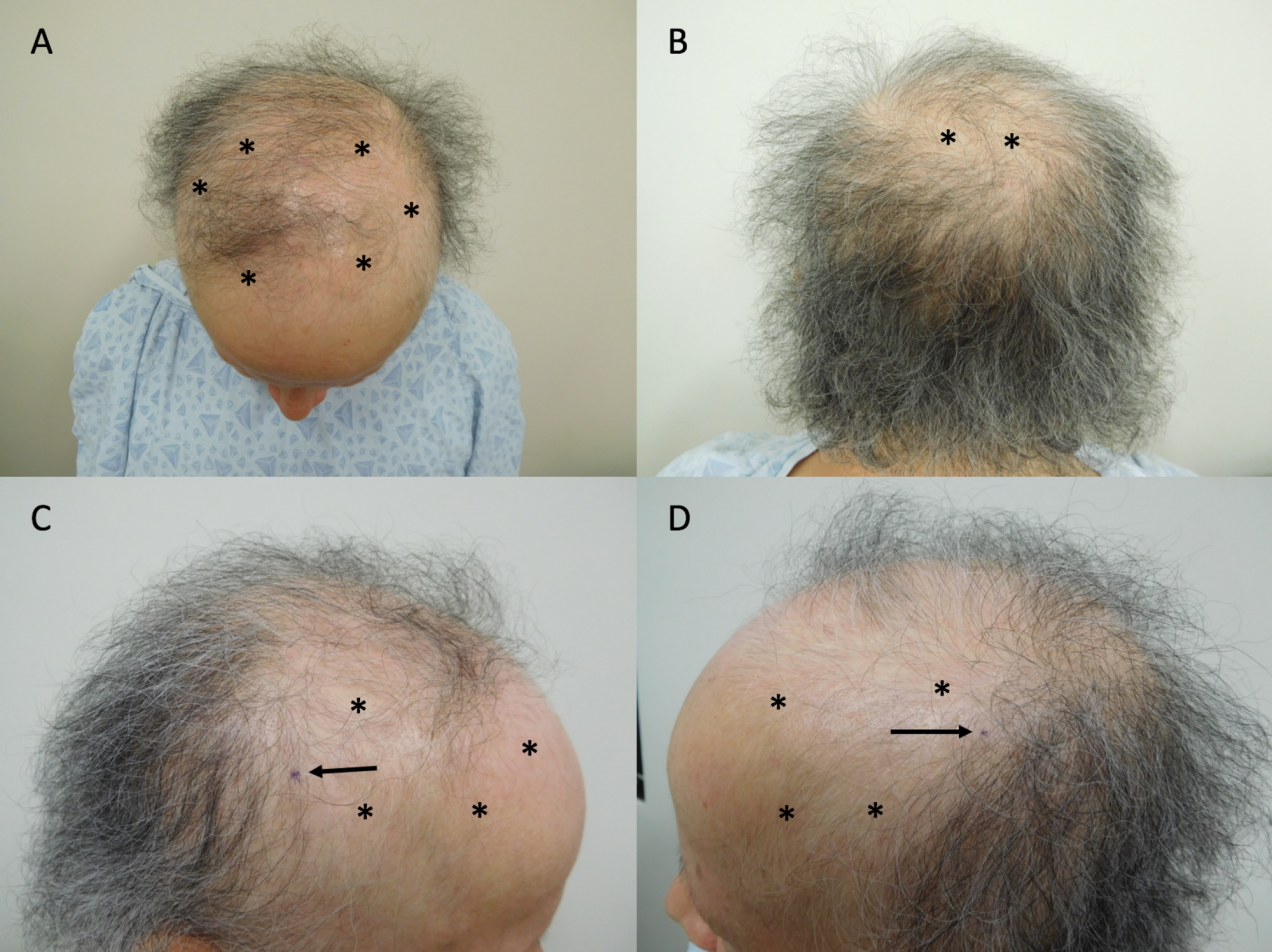
Alopecia in a breast cancer patient after taxane chemotherapy and adjuvant hormonal therapy Top (A), back (B), right (C), and left (D) views of the scalp of a 59-year-old woman with hair loss (*) following taxane (docetaxel) chemotherapy and endocrine therapy (anastrozole followed by tamoxifen) for breast cancer treatment before starting topical minoxidil. The purple dots (arrows) on her right (C), and left (D) scalp are the biopsy sites.

Cutaneous examination revealed alopecia of the scalp. The clinical presentation was most consistent with female pattern alopecia with diffuse and nearly complete hair loss on the central and vertex region with retention of hair on the occipital scalp. There was partial, diffuse hair loss – to a lesser degree – on the parietal scalp bilaterally (Figure 1). There was also loss of hair on the eyebrows, axillae, pubic region, and upper lip. However, these areas had already slowly started to show regrowth.

Biopsies from the right and left sides of her parietal scalp, in areas of alopecia with some preservation of follicles, were performed for horizontal and vertical sectioning. Both showed similar pathologic changes of a non-scarring alopecia. The predominant feature noted was extensive miniaturization of the hair follicles; this change was most suggestive of androgenetic alopecia. However, other findings – present to a lesser extent – included pigment casts in hair follicles, increased catagen to telogen ratio, and empty fibrous tracks; these changes may be observed in alopecia areata.

Correlation of the patient’s history, clinical presentation, and pathologic findings supported a diagnosis of antineoplastic (chemotherapy and hormonal) treatment-associated alopecia. Specifically, her features were consistent with those previously reported in patients with breast cancer after taxane chemotherapy and adjuvant hormonal therapy who developed permanent alopecia [1]. Treatment was initiated with minoxidil 5% foam to be topically applied to the scalp twice daily.

The patient returned for follow up four months later. She was pleased with the clinical outcome and had noticed increased scalp hair growth; however, she commented that she always used minoxidil once daily and occasionally twice daily. In addition, hair growth on the eyebrows, axillae, and pubic area continued to demonstrate clinical improvement. She decided to continue treating her scalp in a similar manner.

**Figure 2:**
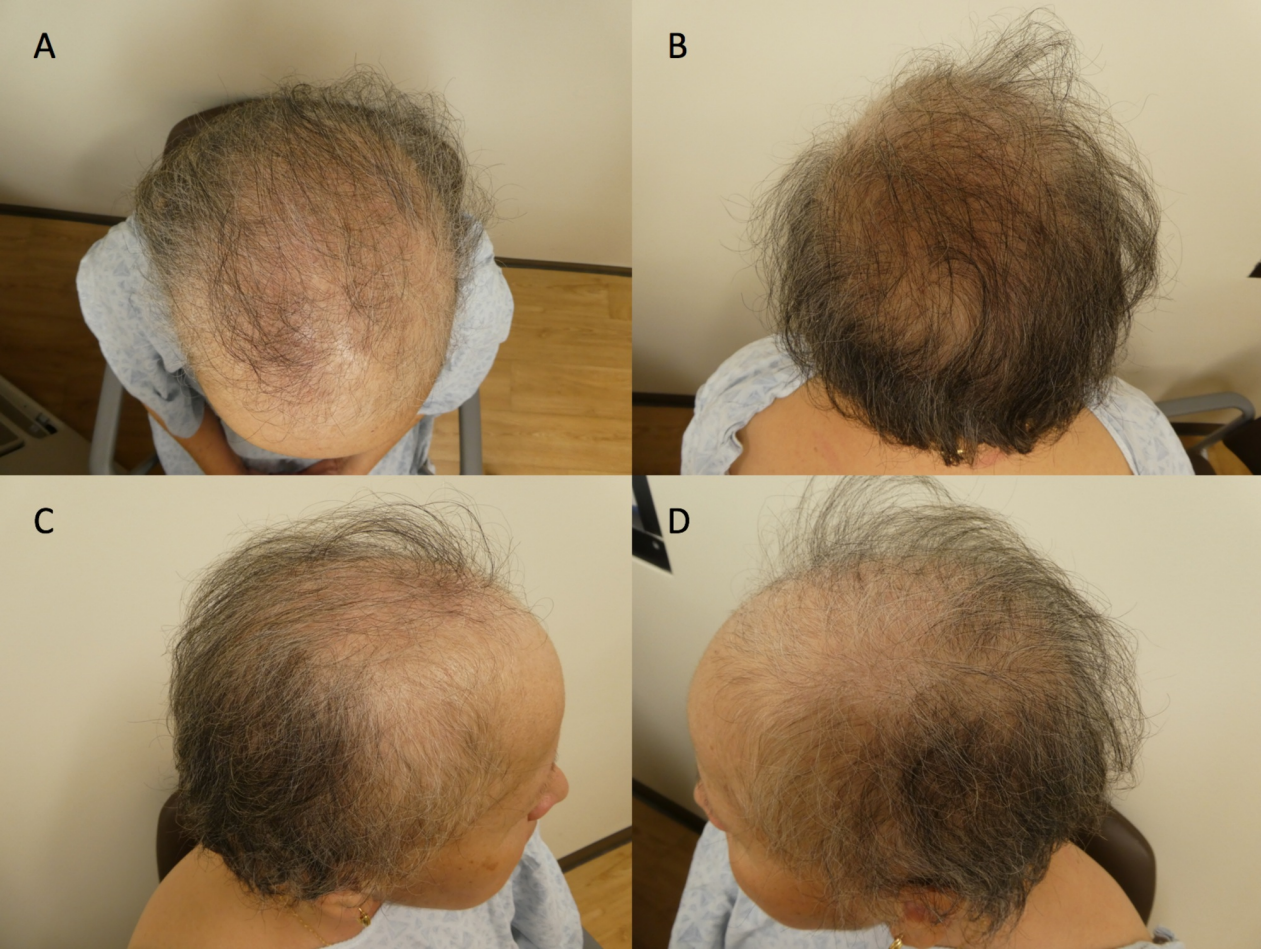
Minoxidil-responsive alopecia following treatment of a breast cancer female patient with taxane chemotherapy and adjuvant hormonal therapy Top (A), back (B), right (C), and left (D) views of the scalp of a 59-year-old woman with partial regrowth of scalp hair after 10 months of topical minoxidil therapy; she had experienced alopecia after her breast cancer treatment which consisted of taxane (docetaxel) chemotherapy and endocrine (anastrozole followed by tamoxifen) therapy.

Her subsequent follow-up visit, six months later (after ten months of topical minoxidil therapy), showed additional hair regrowth. Specifically, the central and vertex area of her scalp had thickening of her hair; in addition, there was new hair growth on the parietal regions bilaterally (Figure 2). She continues to use 5% minoxidil foam once daily.

## Discussion

Alopecia of the scalp is typically classified as scarring or non-scarring. Chemotherapy-induced alopecia is generally non-scarring. Hair loss following treatment with antineoplastic agents is also usually temporary and often presents with an anagen effluvium pattern [2-3].

Various patterns of scalp alopecia have been described in breast cancer patients depending on whether they received chemotherapy, hormonal therapy, or both [4]. In addition to anagen effluvium, patients receiving only endocrine therapy (aromatase inhibitor or selective estrogen receptor modulator) have been observed to develop a pattern similar to androgenetic alopecia [5]. Additionally, permanent alopecia has been observed in some patients who received taxane chemotherapy and adjuvant hormonal therapy; in these individuals, the clinical and pathologic findings were similar to either alopecia areata, androgenetic alopecia or both [1,6].

Our patient’s course of therapy, clinical presentation, and pathologic findings are most consistent with those noted in patients receiving taxane chemotherapy and adjuvant hormonal therapy. The 10 women described by Fonia et al. all had permanent androgenetic alopecia-like hair loss. Two of these women also demonstrated histopathologic findings suggestive of alopecia areata, but only one had patchy hair loss [1].

The report by Fonia et al. did not discuss any treatment interventions [1]. Although our patient is similar to those described in this group, she had a partial improvement of her alopecia following topical intervention with minoxidil 5% foam. The positive therapeutic action of the topical minoxidil may have occurred since the pathologic features of her skin biopsies predominantly corresponded to androgenetic alopecia. Indeed, it is interesting to speculate whether she would have had an even more pronounced hair growth had she been concurrently treated off-label with oral finasteride.

After her initial chemotherapy, while receiving hormonal therapy, she continued to receive trastuzumab 6 mg/kg every three weeks. Trastuzumab is a HER2-specific monoclonal antibody used to treat HER2+ cancers. Cutaneous adverse events associated with this medication are uncommon, and alopecia, in particular, is rarely observed [7]. Rare cutaneous side effects that have been reported in association with trastuzumab include tufted hair folliculitis [8] and psoriasis [9].

Adjuvant endocrine therapy is frequently used in the treatment of hormone receptor-positive breast cancer. Selective estrogen receptor modulators, such as raloxifene, tamoxifen, and toremifene, competitively inhibit estrogen receptors and are preferred for premenopausal women [10]. In addition, leuprolide, a gonadotropin-releasing hormone (GnRH) analog, can be used in premenopausal women in combination with other chemotherapy or endocrine therapy [11]. In comparison, aromatase inhibitors, including anastrozole, exemestane, and letrozole, reduce blood estrogen levels by inhibition of aromatase and are preferred for postmenopausal patients [12]. These treatments have similar adverse event profiles, which include arthralgias, hot flashes, mood changes, and osteopenia [11-12]; alopecia has also been observed [5]. Serious cutaneous adverse effects associated with these therapies are rare, but angioedema, erythema nodosum, porphyria cutanea tarda, pseudolymphoma, radiation recall dermatitis, Steven-Johnson syndrome, subacute cutaneous lupus erythematosus, and vasculitis have been observed [13-17].

In women with breast cancer who developed endocrine therapy-induced alopecia, Freites-Martinez et al. observed significant clinical improvement in 80% (37/46) of the patients after treatment with topical minoxidil [5]. All individuals in this study had not previously received cytotoxic chemotherapy. Our patient considered her improvement with topical minoxidil to be significant. Therefore, it may be reasonable to initiate therapy with 5% minoxidil (foam or solution) twice daily in breast cancer patients who develop alopecia after receiving endocrine therapy alone or following treatment with taxane therapy.

## Conclusions

Chemotherapy-induced alopecia is a frequent side effect of treatment in oncology patients. Breast cancer patients may develop alopecia secondary to their chemotherapy (particularly if it includes a taxane) or adjuvant hormonal therapy or both. Our patient, who was treated with docetaxel and anastrozole followed by tamoxifen, developed persistent alopecia; her clinical presentation and pathology demonstrated a mixed pattern of predominantly androgenetic alopecia with features of alopecia areata. She noted significant, albeit not complete, hair regrowth following treatment with minoxidil 5% foam. It may be reasonable to consider a prospective trial of empiric treatment with topical 5% minoxidil in all breast cancer patients prior to, during, and following their antineoplastic therapy.
